# Alloimmunization in a Rhesus (Rh) D-Negative Pregnant Woman With an Uncommon Rh Phenotype: A Case Report

**DOI:** 10.7759/cureus.86861

**Published:** 2025-06-27

**Authors:** Digeet Davad, Harsh D Majithiya, Jay Nagda

**Affiliations:** 1 Department of Immunohematology and Blood Transfusion, Shri M. P. Shah Government Medical College, Jamnagar, IND; 2 Department of Pathology, Shri M. P. Shah Government Medical College, Jamnagar, IND; 3 Department of Community Medicine, Shri M. P. Shah Government Medical College, Jamnagar, IND

**Keywords:** anti-d antibody, hemolytic disease of the fetus and newborn (hdfn), indirect coombs test, rare rh phenotype, rh alloimmunization

## Abstract

Rhesus (Rh) alloimmunization remains a critical concern in obstetrics, particularly in RhD-negative pregnant women who have not received postpartum Rh immunoglobulin (RhIg) prophylaxis. This report describes the case of a 19-year-old pregnant woman in her second pregnancy who had no documented administration of anti-D prophylaxis after her first childbirth. During routine screening at 28 weeks of gestation, her Indirect Coombs Test (ICT) showed strong positivity (3+), with an anti-D antibody titer of 1:128. Antibody identification confirmed anti-D specificity. Further immunohematological evaluation revealed that the patient had blood group B negative and an uncommon dCe/dce (also known as r′r) Rh phenotype, lacking D and E antigens. Though the fetus was at risk for hemolytic disease of the fetus and newborn (HDFN), there was no need for intrauterine transfusion during the pregnancy. This case highlights the importance of routine antenatal antibody screening, awareness of Rh phenotypes, and timely prophylactic measures to prevent Rh alloimmunization. Comprehensive monitoring and genotype-informed transfusion planning are especially crucial in resource-limited settings.

## Introduction

The Rh blood group system (ISBT: 004) is a cornerstone of transfusion medicine and obstetrics, second only to the ABO system in clinical significance [1]. Central to this system is the D antigen, encoded by the *RHD* gene on chromosome 1p36.11, renowned for its high immunogenicity [2,3]. The *RHE* gene, on the other hand, encodes for C, c, E, and e antigens, which are also clinically relevant, especially in patients who may present with complex serologic profiles.

Individuals who are Rhesus (Rh)-negative, meaning they lack the D antigen on their red blood cells (RBCs), are at risk of developing alloantibodies, particularly anti-D, when exposed to Rh-positive RBCs during events like pregnancy, blood transfusion, or other sensitizing incidents [4]. These alloantibodies can cross the placental barrier, potentially triggering hemolytic disease of the fetus and newborn (HDFN), a serious condition that may result in fetal anemia, hydrops fetalis, or, in severe cases, fetal or neonatal death [5]. In India, the prevalence of RhD-negative individuals ranges from 5% to 8%, with significant regional variation [6]. Routine antenatal screening and postpartum Rh immunoglobulin (RhIg) prophylaxis are not universally practiced across all healthcare levels due to resource limitations. The advent of RhIg prophylaxis has dramatically reduced the incidence of Rh alloimmunization, with data from high-resource countries showing an over 80% reduction in HDFN. However, in low-resource settings like India, these benefits may not fully translate due to gaps in access and implementation [7].

The Indirect Coombs Test (ICT) plays a vital role in identifying alloantibodies in pregnant women, enabling early interventions to safeguard against HDFN [8]. Elevated ICT titers signal significant alloantibody levels, which can complicate perinatal care, often requiring advanced measures like intrauterine transfusions, specialized fetal monitoring, or preterm delivery [9]. Additionally, rare Rh phenotypes, such as the D-- phenotype (lacking RHE-encoded antigens C, c, and e), can further complicate management by producing anti-Rh17 (anti-Hr0) alloantibodies, heightening the risk of hemolytic transfusion reactions or HDFN [10].

In this report, we present a case of a 19-year-old RhD-negative pregnant woman with no documented postpartum RhIg administration in her first pregnancy, who developed alloimmunization due to anti-D antibodies in her second pregnancy. The case highlights diagnostic, phenotypic, and management challenges within a resource-limited setting.

## Case presentation

Patient background

A 19-year-old pregnant woman residing in an urban slum of western India presented for a routine antenatal check-up during her second pregnancy. She had two documented antenatal visits prior to referral. Routine antibody screening is not universal at local primary care clinics, and the patient lived in a low-resource setting with limited access to consistent healthcare, which posed challenges for regular follow-up and documentation. Her obstetric history indicated she was Gravida 2, Para 1, Live 1 (G2P1L1), with a previous full-term normal vaginal delivery 1.5 years prior at a local health facility. No prior screening record and medical records were available to confirm the administration of anti-D immunoglobulin (RhIg) following her first delivery or during the postpartum period, a critical intervention for preventing Rh alloimmunization in RhD-negative women. The patient reported no history of spontaneous or induced abortions, ectopic pregnancies, or blood transfusions, which are potential sensitizing events for Rh alloimmunization.

Her current pregnancy was at 28 weeks and two days of gestation, confirmed by her last menstrual period and corroborated by an early first-trimester ultrasound. She had no significant medical or surgical history, no known allergies, and no history of autoimmune disorders. Her socioeconomic status and limited healthcare access underscored the importance of tailored management to mitigate risks associated with her Rh-negative status and alloimmunization.

Clinical and laboratory evaluation

The patient’s blood group was confirmed as B negative (RhD-negative) through hemagglutination (HA) testing, and the father’s Rh status could not be determined due to resource limitations. Screening for transfusion-transmitted infections, including human immunodeficiency virus (HIV), hepatitis B surface antigen (HBsAg), and hepatitis C virus (HCV), was negative, indicating no additional infectious risks complicating her pregnancy. Her hemoglobin level was 11.2 g/dL, within the normal range for pregnancy (According to the American College of Obstetricians and Gynecologists (ACOG), anemia in pregnancy is defined as a hemoglobin level less than 11.0 g/dL in the first and third trimesters and less than 10.5 g/dL in the second trimester [11]), and her vital signs were stable (blood pressure: 110/70 mmHg, pulse: 82 beats per minute, temperature: 36.8°C).

An obstetric ultrasound performed at 28 weeks and two days revealed a single live intrauterine fetus with appropriate growth parameters for gestational age (estimated fetal weight: 1.2 kg, within the 50th percentile), normal placental position, and an adequate amniotic fluid index (AFI) of 12 cm. Doppler studies of the fetal middle cerebral artery (MCA) peak systolic velocity (PSV) were within normal limits (MCA-PSV: 38 cm/s, <1.5 multiples of the median (MoM)), indicating no evidence of fetal anemia or hydrops fetalis at this stage. Given the high anti-D titer of 1:128, serial fetal surveillance with MCA Doppler was planned on a weekly basis to closely monitor for evolving fetal anemia.

Given her RhD-negative status and history of a prior pregnancy without documented RhIg administration, an ICT was performed to assess for alloimmunization. The ICT yielded a strong positive result (3+), indicating the presence of alloantibodies. Further antibody titration revealed an anti-D antibody titer of 1:128, a level considered high and suggestive of significant alloimmunization. Antibody screening using a three-cell panel showed strong agglutination with Cell I and Cell II (4+), and a negative reaction with Cell III, confirming the specificity of the alloantibody as anti-D. The lack of reactivity with Cell III, which expresses c and E antigens but lacks D, supports the exclusion of anti-c and anti-E alloantibodies, reinforcing the specificity of the detected antibody as anti-D. No additional alloantibodies or autoantibodies were detected, as confirmed by a negative direct antiglobulin test (DAT) and autocontrol (Table 1).

**Table 1:** Clinical and laboratory evaluation ICT: Indirect Coombs Test; EFW: estimated fetal weight; MCA: middle cerebral artery; PSV: peak systolic velocity; MoM: multiples of the median; HBsAg: hepatitis B surface antigen; HCV: hepatitis C virus; GA: gestational age; ACOG: American College of Obstetricians and Gynecologists

Parameter	Patient Value	Normal Reference Range/Notes
Age	19 years	Reproductive age: 15–49 years
Gravida/Para	G2P1L1A0	Gravida 2 = 2 pregnancies; Para 1 = 1 birth; L1 = 1 living; A0 = 0 abortion
Blood Group	B Negative (RhD−)	Blood group: A/B/AB/O; Rh: Positive or Negative
HIV/HBsAg/HCV	Non-reactive	Negative (Non-reactive) is normal
Hemoglobin	11.2 g/dL	Normal in first and third trimesters: ≥11 g/dL; in second trimester: ≥10.5 g/dL (ACOG cutoff)
Ultrasound (GA, Fetal Status)	28+2 weeks, Single live fetus	Consistent with GA
EFW	1.2 kg	Mean EFW at 28 weeks: ~1.0–1.2 kg
Amniotic Fluid Index	12 cm	Normal: 8–18 cm
MCA-PSV	38 cm/s	Normal at 28 weeks: <1.5 MoM ≈ <40 cm/s
ICT	3+	Negative (<1+) is normal; 3+ = strong positive
ICT Titer	1:128	Critical titer: ≥1:16–1:32 (≥1:16 is usually significant)
Antibody Screening (Cell I)	4+	Negative expected; 4+ = strong agglutination
Antibody Screening (Cell II)	4+	Negative expected; 4+ = strong agglutination
Antibody Screening (Cell III)	Negative	Negative expected
Specificity of Alloantibody	Anti-D	Anti-D commonly implicated in Rh isoimmunization
History of Anti-D Administration	Not documented	Important if Rh-negative mother had prior sensitization
History of Blood Transfusion	None	Rule out other causes of alloimmunization

The antigen profiles of the three screening cells (Cell I, II, and III) used to detect anti-D were consistent with the reactivity pattern expected in such alloimmunization cases (see Appendices).

Advanced immunohematological workup

The absence of the Kell antigen (K−) ruled out additional alloimmunization risks related to the Kell blood group system, which can also cause severe HDFN. The negative DAT (both polyspecific and monospecific IgG) and autocontrol further confirmed that the alloimmunization was not complicated by autoimmune hemolytic processes (Table 2).

**Table 2:** Advance immunohematology workup HA: hemagglutination; SPRCA: solid phase red cell adherence; CTT: column tube technique; DAT: direct antiglobulin test; IgG: immunoglobulin G; AHG: anti-human globulin *Both 11-cell panels showed identical reactivity, confirming specificity and reproducibility.

Test Performed	Method	Interpretation
ABO and Rh Blood Grouping	HA	B Negative
Antibody Screening	SPRCA	Positive
DAT (Polyspecific)	CTT	Negative
DAT (Monospecific-IgG)	SPRCA	Negative
Auto Control	CTT	Negative
Antibody Identification by 11-cell panel (AHG phase)*	CTT-Lot id 51918	Positive
Antibody Identification by 11-cell panel (AHG phase)*	CTT-Lot id 43857	Positive
Rh and Kell Phenotyping	HA	C+c+E−e+K−
Presumed Rh Genotype	-	dCe/dce (r′r)

An advanced immunohematological evaluation was conducted to further characterize the patient’s RBC phenotype and presumed Rh genotype. The Rh genotype was presumed based on serologic phenotyping, as molecular genotyping was not performed due to logistical limitations. The results, using hemagglutination and solid-phase red cell adherence (SPRCA) methods, confirmed her RBC phenotype as C+c+E−e+K−, consistent with a presumed Rh genotype of dCe/dce (r′r) (Immucor, Inc., Norcross, Georgia, United States). The absence of D and E antigens on her RBCs, coupled with the presence of C, c, and e antigens, supported her RhD-negative status and the development of anti-D alloimmunization, likely due to exposure during her first pregnancy or delivery (Table 3). The strong reactivity with D-positive cells across the enzyme-treated (ficin) panel and absence of reaction with other Rh antigens confirmed the specificity of anti-D, effectively excluding anti-C, anti-c, or anti-E antibodies.

**Table 3:** Phenotypic evaluation 0: antigen absent; D, C, c, E, e: antigens of the Rh Blood Group System; K: Kell antigen

Blood Group	D	C	c	E	e	K	Genotype (Presumed)
B Negative	0	3+	4+	0	4+	0	dCe/dce(r’r)

The extended red cell panel reactivity using the Panocell®-10 Ficin-treated panels enhances the reactivity of Rh antigens and is used to distinguish anti-D from other antibodies like anti-Fya or anti-Jka (See Appendix B, D).

Fetal monitoring and management

Despite the high anti-D antibody titer (1:128), serial fetal Doppler evaluations performed every one to two weeks showed no evidence of fetal anemia (MCA-PSV consistently <1.5 MoM) or hydrops fetalis (no ascites, pericardial effusion, or skin edema on ultrasound). The critical titer threshold for anti-D antibodies varies by institution but is often considered ≥1:16 or ≥1:32, above which the risk of HDFN increases significantly. Given the titer of 1:128, close monitoring was warranted. The patient was managed with multidisciplinary input from obstetrics, hematology, and fetal medicine specialists. 

The management plan included: (i) Serial ultrasound and Doppler monitoring: Weekly ultrasounds to assess fetal growth, amniotic fluid, and MCA-PSV to detect early signs of fetal anemia; (ii) Counseling: The patient was counseled on the risks of HDFN, the importance of regular follow-up, and the potential need for interventions such as IUT or early delivery if fetal anemia developed; (iii) No IUT: As no signs of fetal anemia or hydrops were detected, IUT was not required during the antenatal period; (iv) Preparation for delivery: The patient was advised to deliver at a tertiary care center equipped with a neonatal intensive care unit (NICU) and blood bank facilities for potential neonatal transfusion or exchange transfusion post-delivery; (v) Postpartum RhIg administration: Although the patient had already developed alloimmunization, RhIg was administered postpartum to reduce further antigenic exposure. Non-invasive fetal RhD genotyping was not performed due to a lack of availability at our center.

Outcome and follow-up

The patient continued antenatal care with regular monitoring. At 36 weeks, an ultrasound confirmed continued fetal well-being, with normal growth and no signs of anemia or hydrops. The patient was admitted at 38 weeks of gestation for planned induction of labor. She delivered a live male neonate via vaginal delivery with a birth weight of 2.8 kg and APGAR scores of 8 and 9 at one and five minutes, respectively. The neonate’s blood group was determined to be B positive, confirming Rh incompatibility.

Postpartum, the neonate was monitored in the NICU for 48 hours. There were no signs of anemia or hyperbilirubinemia necessitating transfusion or phototherapy. The mother received 300 μg of anti-D immunoglobulin within 24 hours of delivery. Although she had already developed alloimmunization, this was administered to reduce further antigenic exposure in subsequent pregnancies. Both mother and baby were discharged in stable condition on day 3 postpartum.

## Discussion

In the Indian population, the prevalence of certain blood group antigens shows distinct patterns. The C antigen (RH2) is found in 83.8% of individuals, while the c antigen (RH3) occurs in 58.1%. The E antigen (RH4) has a lower prevalence at 19.4%, whereas the e antigen (RH5) is highly prevalent at 98.4%. Regarding the Kell blood group system, the K antigen (KEL1) is relatively rare, present in only 4.4% of the population, while the k antigen (KEL2) is almost universally present at 99.9%. These figures suggest that the Indian population exhibits a high prevalence of e, C, and k antigens, similar to other Asian populations, and a notably low frequency of the K antigen (4.4%) compared to Caucasian populations (9%) [12].

Alloimmunization due to anti-D antibodies remains a significant challenge in Rh-negative pregnancies, particularly when anti-D prophylaxis is missed. In our case, a high titer (1:128) was detected, necessitating close fetal monitoring. Studies suggest that titers ≥1:16-1:32 are associated with a significant risk of fetal anemia and hydrops fetalis, especially if rising trends are observed [5,9]. In this context, weekly MCA-PSV Doppler was utilized, as it provides a reliable non-invasive method for detecting fetal anemia. While MCA-PSV Doppler is recommended in cases with titers above the critical threshold, its cost-effectiveness in resource-limited settings remains a concern. However, the risk-benefit ratio favors continued surveillance, given the potentially catastrophic outcomes of undetected HDFN. Guidelines suggest Doppler should be initiated after the critical titer is identified and continued weekly or biweekly, depending on titer trends and gestational age [9,10]. Weekly frequency was chosen based on the stability of the titer and gestational age.

Regarding the timing of delivery, we opted for induction at 38 weeks, a practice supported by other studies indicating that delaying delivery in alloimmunized pregnancies may increase the risk of intrauterine fetal demise even in the absence of MCA Doppler abnormalities [13]. Literature shows that elective delivery between 37-38 weeks can reduce the risk of unexpected fetal decompensation while maintaining favorable neonatal outcomes [12].

Although the patient was already alloimmunized in the present case, postpartum RhIg was administered to minimize further exposure to Rh antigens during future pregnancies, as recommended by some guidelines.

Epidemiologically, studies estimate that among sensitized pregnancies with anti-D titers ≥1:64, approximately 70-80% result in normal outcomes, 15-20% may develop mild anemia, and 2-5% may progress to hydrops fetalis if unmanaged [4,5]. Therefore, the integration of antibody titration, MCA Doppler, and timely delivery forms the cornerstone of contemporary management.

Non-invasive fetal RhD genotyping was not performed due to a lack of availability at our center. The Rh genotype discussed was presumed based on serologic phenotyping since molecular genotyping facilities were not accessible. The ficin-treated red cell panel was employed specifically to enhance reactivity with Rh antigens and help differentiate anti-D from other antibodies like anti-Fya or anti-Jka. The enzyme-enhanced reactions showed strong agglutination with D-positive cells while ruling out reactivity with C, c, and E antigens. This confirmed anti-D specificity and excluded other Rh alloantibodies.

This case emphasizes the importance of structured perinatal strategies, including routine antibody screening, timely RhIg administration, multidisciplinary care, and infrastructure readiness for monitoring and intervention, especially in socioeconomically disadvantaged settings.

## Conclusions

This case highlights the preventable nature of Rh alloimmunization and the severe consequences of missed anti-D prophylaxis. Early detection through routine Rh typing and ICT, coupled with vigilant fetal monitoring and timely interventions, is crucial for managing high-titer cases and ensuring favorable maternal and fetal outcomes. Phenotypic evaluation of blood donors can support inventory management and facilitate urgent matching in sensitized patients.

## Figures and Tables

**Figure 1:**
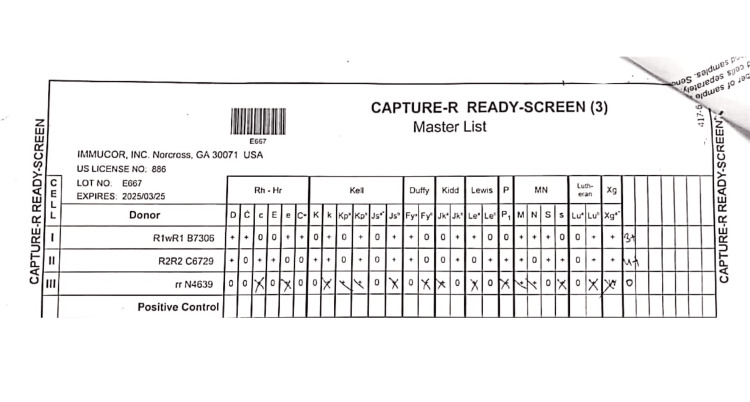
Master List of Antigen Typing Results for Donor Red Blood Cells Using CAPTURE-R Ready-Screen (3) Panel This figure presents the antigen typing profile of three reagent red blood cell donors (Cell I, II, and III), each phenotyped for antigens from Rh, Kell, Duffy, Kidd, Lewis, P, MNS, Lutheran, and Xg systems. Symbols like (+), (0), and (X) denote presence, absence, or non-reactivity of antigens.

**Figure 2:**
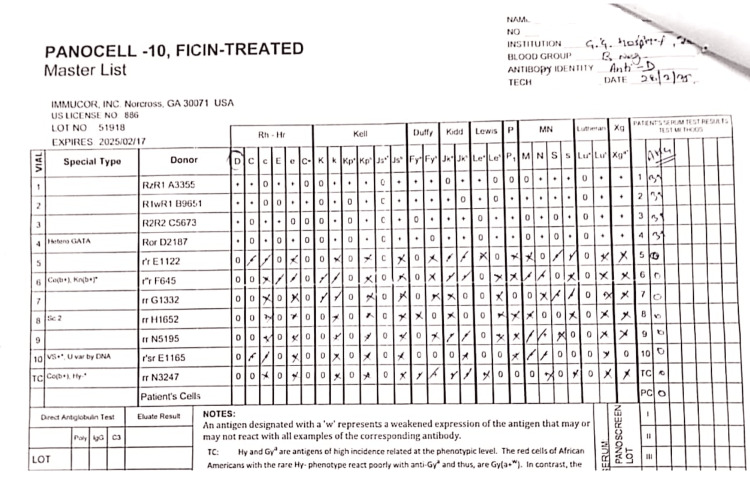
Master List of Antigen Typing Results for PANOCELL-10 Ficin-Treated Donor Red Blood Cells with Corresponding Patient Serum Test Reactions This figure details antigen profiles and corresponding agglutination reactions for eleven reagent red cells, used for improved detection of anti-D after ficin enzyme treatment.

**Figure 3:**
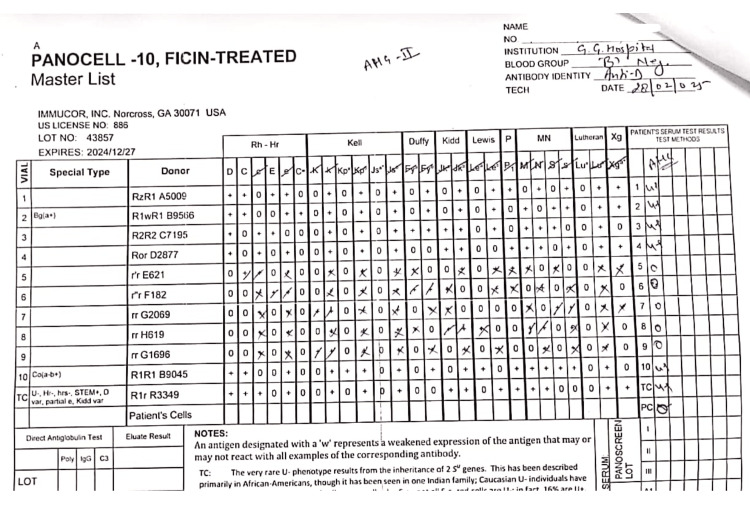
Antigen Typing Results of PANOCELL-10 Ficin-Treated Red Cell Panel with Corresponding Patient Serum Reactions for Anti-D Antibody Identification This panel includes an additional red cell donor and a positive control, illustrating agglutination outcomes, with annotations on weakened and rare antigen expressions critical for serological interpretation.

## Appendices

Appendix A

Appendix B

Appendix C
